# Anti-Sarcopenic Obesity Effects of *Lonicera caerulea* Extract in High-Fat Diet-Fed Mice

**DOI:** 10.3390/antiox10101633

**Published:** 2021-10-17

**Authors:** You-Suk Lee, Eun-Jung Park, Sung-Min Kim, Jong-Yeon Kim, Hae-Jeung Lee

**Affiliations:** 1 Department of Food and Nutrition, Gachon University, Seongnam-si 13120, Gyeonggi-do, Korea; ysleeyun@gachon.ac.kr (Y.-S.L.); ejpark@gachon.ac.kr (E.-J.P.); 2 Institute for Aging and Clinical Nutrition Research, Gachon University, Seongnam-si 13120, Gyeonggi-do, Korea; 3 Department of Food Science and Biotechnology, Gachon University, Seongnam-si 13120, Gyeonggi-do, Korea; kimsm127@gachon.ac.kr (S.-M.K.); whddus95@gachon.ac.kr (J.-Y.K.)

**Keywords:** *Lonicera caerulea*, honeysuckle berry, sarcopenic obesity, high-fat diet

## Abstract

Sarcopenic obesity is a combination of sarcopenia and obesity. Although several herbal extracts showed improvement on sarcopenia and obesity, respectively, there are few studies on sarcopenic obesity. *Lonicera caerulea* (honeysuckle berry, HB) can ameliorate metabolic disorders including obesity. However, its effects on sarcopenic obesity have not been reported yet. Thus, the aim of this study was to investigate whether HB extract might have any beneficial effects on sarcopenic obesity in high-fat diet-induced mice. Forty-eight mice were classified into six groups and treated for eight weeks: (1) NC, normal diet control; (2) HC, high-fat diet control; (3) PC, high-fat diet with orlistat; (4) HB100, high-fat diet with HB extract at 100 mg/kg; (5) HB200, high-fat diet with HB extract at 200 mg/kg; and (6) HB400, high-fat diet with HB extract at 400 mg/kg. Body weight, fat accumulation, muscle mass, muscle strength, and mRNA expression of muscle atrophy were monitored. Compared with the HC group, HB administration showed anti-obesity properties. It reduced body weight gain and modulated serum biochemical parameters and tissue antioxidant enzymes. HB also increased muscle strength and muscle mass of hind legs. In addition, it decreased mRNA expression levels of Atrogin1 and MuRF1 as markers of muscle atrophy but increased PGC1α and SIRT1 as markers of muscle growth. These results suggest that HB might be effective in preventing sarcopenia associated with obesity.

## 1. Introduction

Sarcopenic obesity refers to reduced skeletal muscle mass and muscle strength in parallel with an increase of body fat [1]. Excessive body fat causes muscle atrophy by suppressing protein synthesis in skeletal muscles [2]. Increased fat mass can impair the oxidative capacity of mitochondria in skeletal muscles, leading to insulin resistance [2,3,4,5]. It has been reported that sarcopenic obesity is associated with an increase in metabolic disease, cardiovascular morbidity and mortality [6,7].

In animal studies, high-fat diet (HFD) induced obesity mice showed muscle atrophy with decreased muscle mass and muscle strength [8,9,10]. Muscle RING-finger 1 (MuRF1) and atrogin1 as markers of skeletal muscle atrophy are expressed early in the atrophy progress. They are involved in the ubiquitin proteasome pathway prior to the loss of muscle mass [11,12,13]. Sirtulin1 (SIRT1) is involved in the regulation of metabolic homoeostasis and mitochondrial function. It can increase oxygen consumption through deacetylation of peroxisome proliferator-activated receptor coactivator 1α (PGC1α) [14].

*Lonicera caerulea* (honeysuckle berry, HB) is grown in cold temperate zones and wet regions in Korea, Europe, North America, Russia, and Japan. It is used as a food and traditional medicine [15,16]. HB has been reported to have various pharmacological activities such as antioxidant, neuroprotective, and hepatoprotective activities [17,18,19]. In our previous study, HB extract tends to increase mRNA levels of anti-oxidant genes such as heme oxygenase-1 (HO-1), NAD(P)H dehydrogenase [quinone] 1 (Nqo1), and glutamate-cysteine ligase catalytic subunit (Gclc) in HepG2 cells. These anti-oxidant genes, which contain anti-oxidant response elements (AREs) in their promoter regions, can be induced by Nrf2 activation. Activities of anti-oxidant enzymes such as superoxide dismutase (SOD) and catalase (CAT) activities were also increased by treatment with HB extract [20].

Fat accumulation in skeletal muscles, which leads to impaired fatty acid oxidation function, increases oxidative stress, pro-inflammatory cytokine production, and macrophages activities, resulting in decline in muscle mass and strength [21]. In the present study, we hypothesized that HB with antioxidant and anti-inflammatory properties may increase muscle mass and strength against sarcopenic obesity.

HB is rich in nutritional and bioactive components, mainly anthocyanins, flavanols, and phenolic acid [17,18,19,22,23]. Due to its rich nutritional components, HB has been reported to be one of the most economically important species belonging to genus *Lonicera L.* [24]. Although a number of studies have reported that HB has anti-oxidant and anti-inflammatory effects, studies regarding its effect on sarcopenic obesity have not been reported to our best knowledge. Therefore, the objective of this study was to assess whether HB extract might have a preventive effect on sarcopenic obesity in HFD-induced obese mice.

## 2. Materials and Methods

### 2.1. Preparation and HPLC Analysis of HB Extract

Raw HB fruits were purchased from Mulsolisaesoli farm, Gangwon-do, Korea and HB extract was prepared as described previously [17] with some modifications. Briefly, Raw HB fruits were finely ground with a blender (Ninja blender DUO, Needham, MA USA) and mixed with deionized water containing 1% citric acid (Sigma-Aldrich, St. Louis, MO, USA) at a ratio of 1:10 (*w/v*). After incubating at 60 °C for 3 h in a water bath (C-WBE, Chang Shin Scientific Co., Seoul, Korea), the mixture was filtered using No. 2 filter paper (Advantec, Toyo Roshi Kaisha, Tokyo, Japan) and concentrated with a rotary vacuum evaporator (EYELA, N-N series, Tokyo, Japan) at 60 °C. The HB extract was stored at −20 °C until further use. Its yield was 25.6%. HPLC analysis was performed to measure the content of cyanidin 3-*O*-glucoside (C3G), an anthocyanin mainly contained in HB [24], using a previously reported method [25] with slight modifications. Briefly, raw HB and HB extract were subjected to ultrasonic-extraction with 70% MeOH containing 0.1% formic acid for 10 min and then filtered with a 0.2 μm syringe filter. Chromatography was carried out on a Sunfire C18 column (4.6 mm × 250 mm, particle size 5 μm) that was maintained at 35 °C using an Agilent 1100 DAD system. The flow rate was 1.0 mL per min. The injection volume and the detector wavelength were set to be 10 μL and 517 nm, respectively. A gradient system consisted of a mobile phase A (water containing 0.1% formic acid) and a mobile phase B (acetonitrile containing 0.1% formic acid).

### 2.2. Animals and Treatment

**Figure 1:**
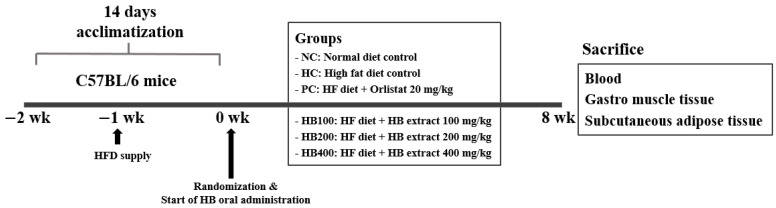
Experimental design of the study.

Male C57BL/6 mice (six-week-old) were purchased from Orient Bio Co. Ltd. (Uiwang-si, Gyeonggi-do, Korea) and acclimated for one week under standard conditions (temperature of 20–25 °C, humidity of 50–55%, and a 12-h light-dark cycle) with free access to food and water *ad libitum*. After the acclimation period, mice were randomly divided into six groups (*n* = 8 per group): (1) NC, normal diet (10% calories from fat; D12450B; Research Diets, New Brunswick, NJ, USA) control; (2) HC, high-fat diet (45% calories from fat; D12451; Research Diets, New Brunswick, NJ, USA) control; (3) PC, high-fat diet with orlistat 20 mg, TCI, Tokyo, Japan); (4) HB100, high-fat diet with HB extract at 100 mg/kg; (5) HB200, high-fat diet with HB extract at 200 mg/kg; and (6) HB400, high-fat diet with HB extract at 400 mg/kg. NC and HC groups of mice were orally given 0.5% water solution of carboxymethyl cellulose sodium (CMC-Na, TCI, Tokyo, Japan) only, while PC and the three HB groups of mice were administrated with an equal volume of the suspended or dissolved test material in 0.5% CMC-Na solution once a day for eight weeks. Schedule of the animal experiment is shown in Figure 1. Body weight was measured once a week. All animals were cared and used in accordance with the guidelines of Gachon University for the care and use of laboratory animals (reference number: GIACUC-R2020009). After 12 h of fasting, all mice were anesthetized with CO_2_. Blood samples were collected by cardiac puncture and clotted in serum-separating tube before centrifuging at 2000× *g* for 20 min at 4 °C. Serum was aliquoted and stored at −80 °C until use. Adipose tissue and gastrocnemius muscles were immediately dissected from mice, weighted, and stored at –80 °C until analysis. The experimental design is shown in Figure 1.

### 2.3. Biochemical Assays

Serum triglyceride (TG) and total cholesterol (TC) levels were measured using appropriate commercial assay kits (Asanpharm, Hwaseong, Korea). Serum adiponectin and leptin levels were analyzed using enzyme-linked immunosorbent assay (ELISA) kits (R&D systems, Minneapolis, MN, USA).

### 2.4. Histological Assay

Gastrocnemius muscle samples were fixed with 10% formalin solution (Sigma-Aldrich, St. Louis, MO, USA) and then processed for paraffin embedding. These samples were stained with hematoxylin and eosin (H&E) and observed under an Olympus Provis AX70 microscope (Olympus, Tokyo, Japan). Images were captured using a Nikon DS-Ri2 camera (Nikon, Tokyo, Japan) with NIS-Elements BR 4.50.00 software (Nickon, Tokyo, Japan). Adipocyte and muscle sizes were quantified using ImageJ-pro10 software (https://imagej.nih.gov/ij/index.html, accessed on 2 September 2021).

### 2.5. Micro-Computed Tomography (Micro-CT) and Muscle Strength

Mice were anesthetized with Zoletil (40 mg/kg) and rompun (5 mg/kg) by intraperitoneal injection (i.p.). Body fat and muscles of hind legs of each mouse were scanned at an isotropic voxel size of 100 µm (45 kVp, 177 µA, 160 ms integration time) with a viva CT 80 scanner (Scanco Medical, Brüttisellen, Switzerland). Two-dimensional gray-scale image slices were reconstructed with three-dimensional tomography. The measured body fat volume included the volume of the abdominal fat and that of the subcutaneous fat in the space from the first thoracic vertebra to the fourth sacrum vertebra. The muscle volume measured the muscle of tibia in the hind limb. Reconstruction of micro-CT images was performed using µCT Evaluation Program V6.6 (Scanco Medical, Brüttisellen, Switzerland). Muscular strength of mouse was also measured using a grip strength meter.

### 2.6. Real-Time PCR Analysis

Total RNA was isolated from adipose tissue and gastrocnemius muscle using a RNA extraction kit (iNtRON Biotechnology, Seongnam-si, Gyeonggi-do, Korea). cDNA was synthesized from RNA (50 ng/μL) using a GoScript™ Reverse Transcriptase (Promega, Madison, WI, USA) and used for real-time RT-PCR with a SYBR Premix Ex Taq II (Takara, Otsu, Japan) using an ABI QuantStudio 3 (Applied Biosystems, Foster City, CA, USA). Primer sequences used for RT-PCR are displayed in Table 1. All gene expressions were normalized to that of β-actin or 18S rRNA (gene name Actb and Rn18s, respectively).

### 2.7. Statistical Analysis

All data are presented as mean ± standard deviation (SD). Statistical differences were determined with one-way analysis of variance (ANOVA) followed by Duncan’s multiple range test. Two-group comparisons were carried out with Student’s t-test using SPSS 25 (SPSS Inc., Chicago, IL, USA). Values of *p* < 0.05 were considered statistically significant.

## 3. Results

### 3.1. Detection of C3G Contents in Raw HB and HB Extract

**Figure 2:**
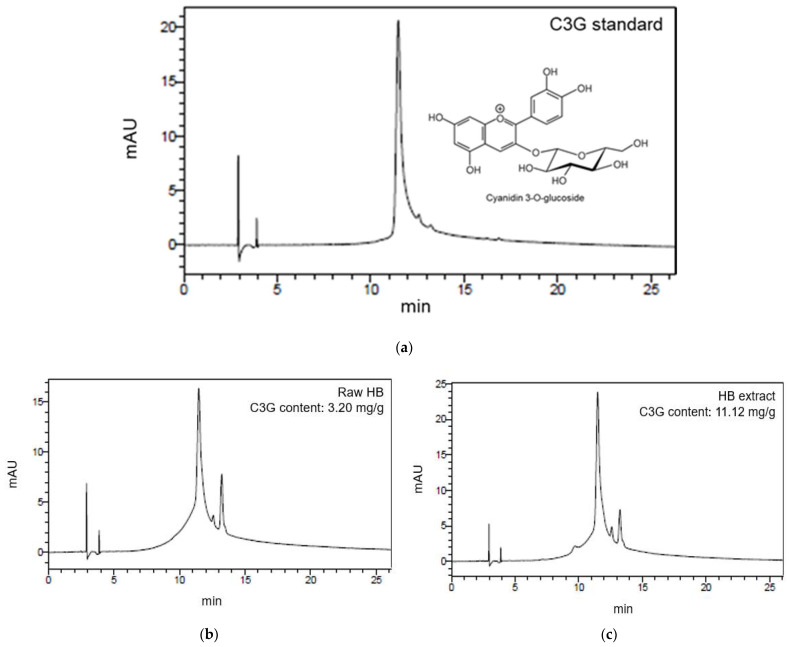
High performance liquid chromatography (HPLC) analysis results. (**a**) C3G standard; (**b**) Raw HB; and (**c**) HB extract.

To determine C3G contents in raw HB and HB extract, HPLC analysis was performed. The main indicator and functional compound of HB extract is C3G, one of the anthocyanin HB [24]. As shown in Figure 2, the C3G content of HB extract was approximately three times higher than that of raw HB (11.12 mg/g and 3.20 mg/g, respectively).

### 3.2. Effects of HB on Body Weights and Serum Biochemical Parameters

**Figure 3:**
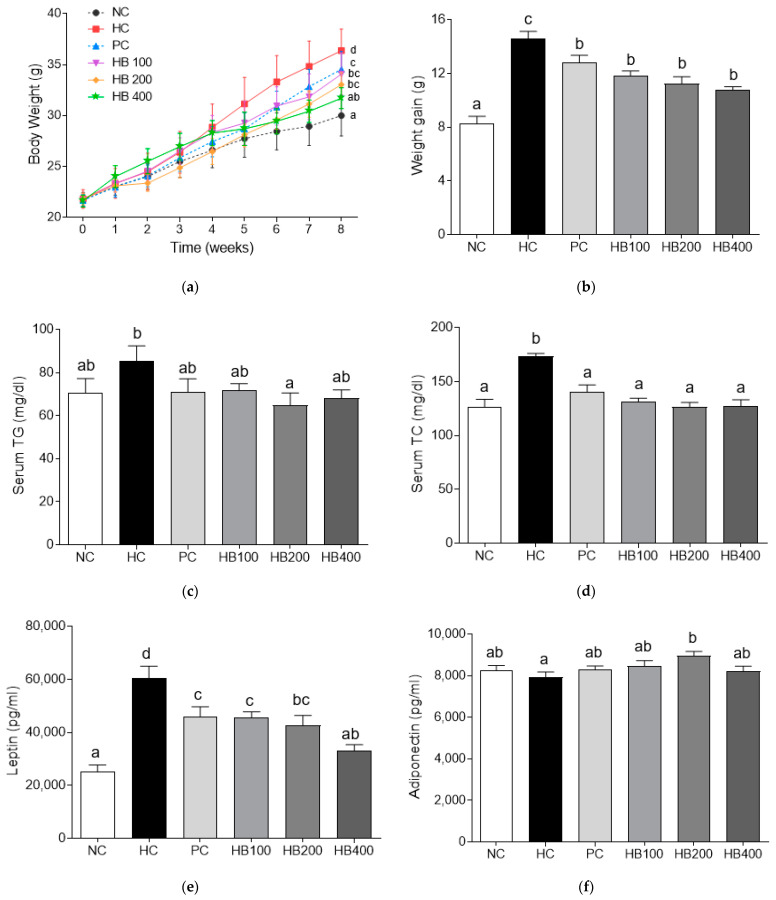
Effects of HB extract on body weight changes and serum biochemistry parameters. (**a**) Body weight; (**b**) weight gain; (**c**) serum TG; (**d**) serum TC; (**e**) leptin; and (**f**) adiponectin. NC, Normal diet Control; HC, HF diet control; PC, HF diet with orlistat 20 mg/kg; HB100, HF diet with honeysuckle berry extract at 100 mg/kg; HB200, HF diet with honeysuckle berry extract at 200 mg/kg; HB400, HF diet with honeysuckle berry extract at 400 mg/kg. Data are presented as mean ± SD (*n* = 8). Different letters indicate significant differences by one-way ANOVA (*p* < 0.05) followed by Duncan’s multiple range tests.

As shown in Figure 3a, there was no significant difference in the initial body weight among groups. However, HFD-fed mice gained more body weights than mice in the NC group at the terminal point. Body weight gains of mice in HB 100, 200, and 400 groups were lower than those of mice in the HC group. Body weight gain was decreased by HB in a concentration-dependent manner (Figure 3b). Effects of HB on serum parameters such as TG, TC, adiponectin, and leptin were also investigated. As shown in Figure 3c–f, the HC group showed higher serum TG, TC, and leptin levels than HB groups. Adiponectin levels of the HC group were lower than that of NC group, while they were significantly increased in the HB200 group.

### 3.3. Effects of HB on Fat Accumulation by Micro-CT Analysis

**Figure 4:**
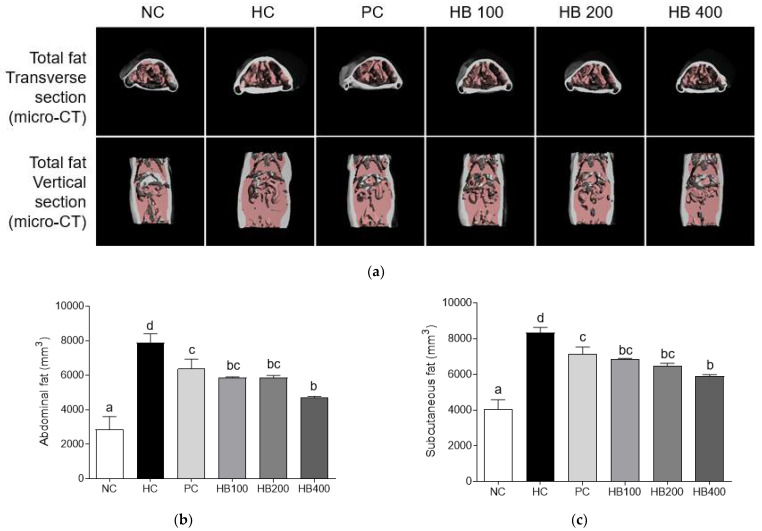
Effects of HB extract on fat mass. (**a**) Micro-CT analysis of total fat transverse section and total fat vertical section; (**b**) abdominal fat volumes; and (**c**) subcutaneous fat volumes. NC, Normal diet Control; HC, HF diet control; PC, HF diet with orlistat at 20 mg/kg; HB100, HF diet with honeysuckle berry extract at 100 mg/kg; HB200, HF diet with honeysuckle berry extract at 200 mg/kg; HB400, HF diet with honeysuckle berry extract at 400 mg/kg; Micro CT, micro-computed tomography. Data are presented as mean ± SD (*n* = 8). Different letters indicate significant differences by one-way ANOVA (*p* < 0.05) followed by Duncan’s multiple range tests.

We analyzed micro-CT images to investigate the effect of HB on fat deposition and abdominal and subcutaneous fat volumes. As indicated in the pink area shown in the photographs of micro-CT (Figure 4a), fat deposition volume of the HC group in both transverse section and vertical section were higher than those of other groups. HB fed groups showed significantly reduced abdominal and subcutaneous fat volumes compared to the HFD only fed group (Figure 4b,c).

### 3.4. Effects of HB on Antioxidant Enzymes

**Figure 5:**
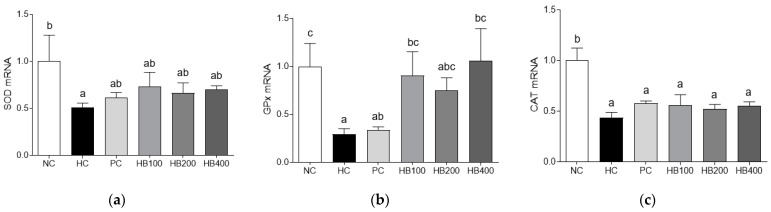
Effects of HB extract on mRNA expression levels of antioxidant enzymes. (**a**) SOD, superoxide dismutase; (**b**) GPX, glutathione peroxidase; and (**c**) CAT, catalase. Gene expressions were normalized to that of β-actin. NC, Normal diet Control; HC, HF diet control; PC, HF diet with orlistat at 20 mg/kg; HB100, HF diet with honeysuckle berry extract at 100 mg/kg; HB200, HF diet with honeysuckle berry extract at 200 mg/kg; HB400, HF diet with honeysuckle berry extract at 400 mg/kg. Data are presented as mean ± SD (*n* = 8). Different letters indicate significant differences by one-way ANOVA (*p* < 0.05) followed by Duncan’s multiple range tests.

We assessed the expression of antioxidant enzymes. Results of mRNA expression analysis of antioxidant enzymes in high fat-fed mice revealed decreased expression levels of SOD, GPX, and CAT enzymes in the HC group than in the NC group, but increased expression levels in HB treated groups than in the HC group (Figure 5a–c).

### 3.5. Effects of HB on Muscle Mass by Micro-CT Analysis

**Figure 6:**
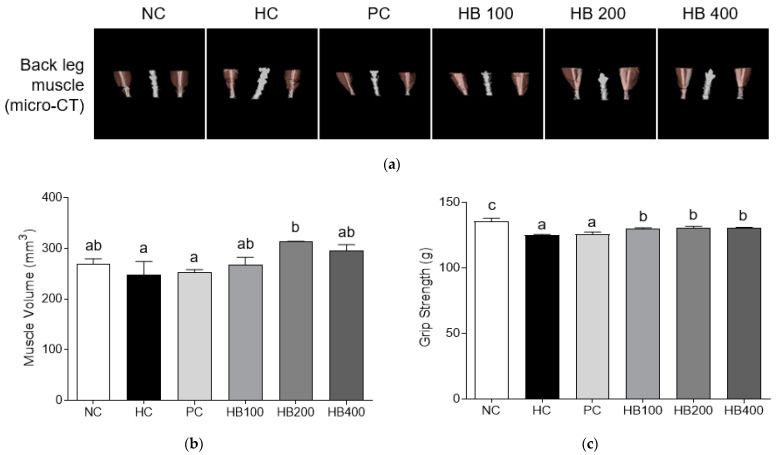
Effect of HB extract on muscle mass. (**a**) micro-CT analysis of hind limb muscle; (**b**) muscle volume; and (**c**) grip strength. NC, Normal diet Control; HC, HF diet control; PC, HF diet with orlistat at 20 mg/kg; HB100, HF diet with honeysuckle berry extract at 100 mg/kg; HB200, HF diet with honeysuckle berry extract at 200 mg/kg; HB400, HF diet with honeysuckle berry extract at 400 mg/kg. Data are presented as mean ± SD (*n* = 8). Different letters indicate significant differences by one-way ANOVA (*p* < 0.05) followed by Duncan’s multiple range tests.

To evaluate the effect of HB on muscle mass and strengths of hind limbs and gastro muscles, we performed a micro-CT analysis (Figure 6a). Results of measuring the volume and muscle deposition using micro-CT system revealed that the volume of hind limb muscle was increased in HB200 and HB400 groups (Figure 6b). As shown in Figure 6a, muscle deposition was especially increased in the HB 200 group. The grip strength was significantly lower in the HC group than in all HB groups (Figure 6c).

### 3.6. Effects of HB on Muscle Mass and Expression Levels of Atrophy Related Genes

**Figure 7:**
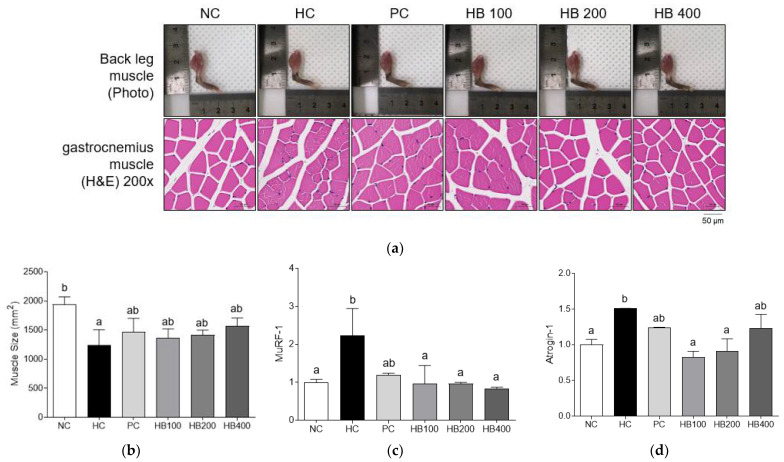
Effects of HB extract on muscle sizes and mRNA expression levels of muscle metabolism related genes. (**a**) Photography of hind limb muscle and H&E staining of gastrocnemius; (**b**) muscle size; (**c**) mRNA expression level of MuRF-1 in the muscle tissue; and (**d**) mRNA expression level of Atrogin-1 in the muscle tissue. Gene expressions were normalized to that of 18S rRNA. NC, Normal diet Control; HC, HF diet control; PC, HF diet with orlistat at 20 mg/kg; HB100, HF diet with honeysuckle berry extract at 100 mg/kg; HB200, HF diet with honeysuckle berry extract at 200 mg/kg; HB400, HF diet with honeysuckle berry extract at 400 mg/kg; Data are presented as mean ± SD (*n* = 8). Different letters indicate significant differences by one-way ANOVA (*p* < 0.05) followed by Duncan’s multiple range tests.

To evaluate the effect of HB on muscle mass and sizes of the hind limbs and gastro muscles, we performed a visual examination and H&E staining analysis (Figure 7a). Visual inspection analysis showed that the hind limb size was not clearly different among groups, although the hind limb sizes of HB200 group were bigger than those of other groups. Skeletal muscle atrophy is characterized by reduction in muscle mass and diameter of muscle fiber [26]. Histological examination results of gastrocnemius cross sections showed that the diameters of muscle fiber were greater in NC and HB groups than those of HC group. Muscle sizes were also lower in the HC group than in other groups. These differences were significant (Figure 7b).

We investigated molecular mechanisms of HB on muscle atrophy. There was a significant up-regulation of mRNA expression of MuRF-1 in the HC group than in HB100, HB200, HB400, and NC groups. In addition, mRNA expression of Atrogin-1 was down-regulated in HB100, HB200, and HB400 groups than in the HC group (Figure 7c,d).

### 3.7. Effects of HB on Growth Related Genes Expression

**Figure 8:**
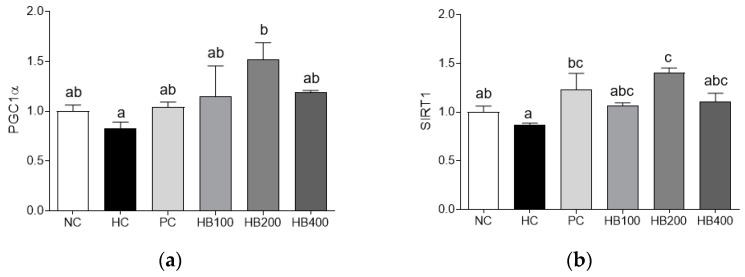
Effects of HB extract on mRNA expression of levels of muscle growth related genes in muscle tissues. (**a**) PGC1α; (**b**) SIRT1. Gene expressions were normalized to that of 18S rRNA. NC, Normal diet Control; HC, HF diet control; PC, HF diet with orlistat at 20 mg/kg; HB100, HF diet with honeysuckle berry extract at 100 mg/kg; HB200, HF diet with honeysuckle berry extract at 200 mg/kg; HB400, HF diet with honeysuckle berry extract at 400 mg/kg. Data are presented as mean ± SD (*n* = 8). Different letters indicate significant differences by one-way ANOVA (*p* < 0.05) followed by Duncan’s multiple range tests.

We also investigated effects of HB on growth related genes in muscle tissues. PGC1α mRNA expression level was reduced in the HC group than in other groups. A different muscle growth related gene, SIRT1, was also down-regulated at mRNA expression level in the HC group than in other groups. HC and HB200 groups showed significant differences in mRNA expression levels of PGC1α and SIRT1 (Figure 8a,b).

## 4. Discussion

Although previous reports have shown health functional properties of HB, the effect of HB on sarcopenic obesity has not been reported yet. In the present study, we confirmed the anti-sarcopenic obesity effect of HB. When fat accumulation and muscle atrophy were checked using micro-CT and H&E staining, HB treatment was observed to alleviate fat accumulation and inhibit muscle atrophy of gastro muscles in the hind limb. In addition, oral administration of HB reduced serum levels of TG and TC in HFD-fed obese mice, indicating that it could attenuate lipid metabolism dysfunction caused by HFD. To the best of our knowledge, this study is the first demonstration of the anti-sarcopenic obesity of HB extract in high-fat diet-fed obese mice. In most parameters, it was effective at all concentrations of HB extract tested. In the case of TG, adiponectin and muscle volume were significantly increased in HB200 group. These results were similar to results after treatment with orlistat, a positive control.

One study has reported that anthocyanin cyanidin-3-O-glucoside (C3G) is the most abundant in HB [24]. Average total anthocyanin content of HB is higher than that of other berries such as blueberry, blackberry, and raspberry [20]. The existence of C3G in HB might explain for its significantly increased antioxidant activity and other biological activities [20]. In the present study, the amount of C3G in raw material of HB was measured to be 3.19 mg/g, whereas it was 11.12 mg/g in water extract containing 1% citric acid. The higher C3G content in the water extract might be due to the use of citric acid that can destroy cell walls in vacuoles, thus increasing C3G content [27].

Among adipokines, adiponectin, and leptin are produced exclusively by adipocytes. Adiponectin is associated with insulin resistance and dyslipidemia. Leptin can increase fatty acid oxidation [28], inhibit appetite [29], stimulate thermogenesis [30]. With the accumulation of fat, the adiponectin level is decreased, the leptin level is increased, and the insulin action is impaired, resulting in insulin resistance [31]. In present results, HB dose-dependently suppressed serum concentrations of leptin but elevated serum concentrations of adiponectin in HFD-fed obese mice.

Excessive fat accumulation is associated with oxidative stress. It can affect obesity and obesity-related syndromes [32]. Activities of antioxidant enzymes such as GPx and SOD are dysregulated in obese individuals compared to those in controls [33]. In the present study, it was found that adipocytic mRNA expression levels of SOD, GPx, and CAT were increased in HB-treated groups. Taken together, these results suggest that HB can prevent fat accumulation (obesity) by regulating the antioxidants enzyme defense system.

Increased body fat can result in muscle loss and atrophy, leading to sarcopenic obesity. Recent studies have indicated that muscle loss and dysfunction are associated with obesity [34,35]. In the present study, results of micro-CT and H&E staining analysis revealed that HB prevented obesity-induced decreases of muscle mass and fiber size in skeletal muscles of HFD-fed mice. Similar to these results, expression levels of muscle RING-finger 1 (MuRF1) and atrogin1 involved in muscle atrophy procession [11] were downregulated by HB, indicating that HB could protect against obesity-induced muscle atrophy. SIRT1 plays a critical role in energy metabolism and oxidative stress by deacetylating target proteins in muscle tissues [36]. Excess energy intake causes activation of caspase I as a part of the inflammasome, which cleaves SIRT1 in adipose and muscle tissues [36]. Sirtulin1 (SIRT1) regulates metabolic homeostasis through deacetylation of peroxisome proliferator-activated receptor coactivator 1α (PGC1α) [14,37]. In this study, expression levels of SIRT1 and PGC1α related to muscle growth were upregulated by HB, resulting in prevention of muscle loss. Our results suggest that HB has potential to be developed as an anti-sarcopenic obesity agent since administration of HB could ameliorate muscle atrophy and upregulate muscle growth-related genes.

This study was limited by the fact that HB contains various bioactive agents, necessitating the isolation and identification of its active compounds, and further experimental studies are required to be conducted using the isolated active compounds.

## 5. Conclusions

HB significantly reduced body weight gain, adipocyte size, and abdominal and subcutaneous fat mass. Expression levels of SOD, GPx, and CAT were upregulated by HB treatment. HB also ameliorated grip strength muscle mass and size by promoting muscle growth related genes SIRT1 and PGC1α while inhibiting muscle atrophy related genes MuRF1 and Artogen1. Our results provide a scientific basis for preventing sarcopenic obesity by HB in high-fat diet-fed obese mice. It is expected that HB may be used as health functional substance to manage sarcopenic obesity. Further clinical studies are needed to confirm the anti-sarcopenic obesity of HB extract demonstrated in this study.

## Figures and Tables

**Table 1:** Primer sequences used for quantitative real-time RT-PCR.

Genes	Accession Number	Forward Primer (5′–3′)	Reverse Primer (5′–3′)
Sod1	NM_011434.2	GTGATTGGGATTGCGCAGTA	TGGTTTGAGGGTAGCAGATGAGT
Gpx1	U13705.1	GAAGAACTTGGGCCATTTGG	TCTCGCCTGGCTCCTGTTT
Cat	NM_009804.2	TGAGAAGCCTAAGAACGCAA	CCCTTCGCAGCCATGTG
MuRF1	NM_001039048.2	GACAGTCGCATTTCAAAGCA	AGGGATTCGCAGCCTGGAAG
Atrogin1	NM_026346.3	CAGCTTCGTGAGCGACCTC	GGCAGTCGAGAAGTCCAGTC
Ppargc1a	NM_008904.2	ATGTGTCGCCTTCTTGCTCT	ATCTACTGCCTGGGGACCTT
Sirt1	NM_019812.3	GCTGACGACTTCGACGACG	TCGGTCAACAGGAGGTTGTCT
Actb	NM_007393.5	CCACAGCTGAGAGGGAAATC	AAGGAAGGCTGGAAAAGAGC
Rn18s	NR_003278.3	AGCCTGAGAAACGGCTACC	TCCCAAGATCCAACTACGAG

Real-time RT PCR, real-time reverse transcription polymerase chain reaction; Sod, superoxide dismutase; Gpx, glutathione peroxidase; Cat, catalase; MuRF1, muscle Ring-finger protein-1; Sirt1, sirtulin1; Ppargc, Peroxisome proliferator-activated receptor-gamma coactivator; Actb, β-actin; Rn18s, 18S rRNA.

## Data Availability

Data is contained within the article.
